# Self-determination, premenstrual syndrome, and health-promoting lifestyle as factors influencing quality of life among Korean female nursing students: a cross-sectional study

**DOI:** 10.7586/jkbns.25.087

**Published:** 2026-02-27

**Authors:** Jiyoung Park, Yunmi Kim

**Affiliations:** 1 Department of Nursing, Presbyterian Medical Center–Jesus Hospital, Jeonju, Korea; 2 Graduate Student, College of Nursing, Jesus University, Jeonju, Korea; 3 College of Nursing, Jesus University, Jeonju, Korea

**Keywords:** Life style, Personal autonomy, Premenstrual syndrome, Quality of life, Students, Nursing

## Abstract

**Purpose:**

This study examined the associations of self-determination (SDT), premenstrual syndrome (PMS), health-promoting lifestyle (HPL), and satisfaction-related factors with quality of life (QoL) among nursing students.

**Methods:**

This cross-sectional descriptive study included 206 nursing students engaged in clinical practice, recruited from nine nursing colleges in Jeollabuk-do, Korea. Data were collected between July 1 and August 19, 2024. Descriptive statistics, independent t-tests, one-way analysis of variance, Pearson correlation analysis, and multiple regression analysis were performed using SPSS version 26.0. SDT, PMS, HPL, satisfaction with economic status, and QoL were assessed using validated self-report questionnaires.

**Results:**

Factors significantly associated with QoL included HPL (β = .37, *p* < .001), SDT (β = .24, *p* < .001), PMS (β = −.21, *p* < .001), menstrual quantity (average vs. large) (β = .13, *p* = .028), satisfaction with nursing major (satisfied vs. dissatisfied) (β = −.11, *p* = .035), and satisfaction with economic status (satisfied vs. dissatisfied) (β = .16, *p* = .001). The overall regression model was statistically significant (F = 27.56, *p* < .001) and explained 61% of the variance in QoL.

**Conclusion:**

From a biological nursing perspective, physiological factors such as PMS and menstrual quantity, together with HPL and SDT, were significant correlates of QoL among nursing students, alongside satisfaction with academic major and economic status. These findings suggest that nursing education programs should promote HPL and SDT while providing systematic support for PMS management and economic stress reduction to enhance students’ QoL.

## INTRODUCTION

### 1. Background

Nursing students represent the future workforce of the healthcare system, and their quality of life (QoL) during the academic transition is a critical predictor not only of their individual well-being but also of the quality of care they will eventually provide [1,2]. QoL is regarded as a multidimensional construct that reflects individuals’ perceptions of their overall well-being, including physical health, psychological state, social relationships, and their ability to function in daily life and is shaped by diverse personal and contextual factors among female nursing students [3]. However, maintaining optimal QoL is particularly challenging for nursing students due to a rigorous curriculum that combines intense academic coursework with clinical rotations [1]. Junior (3rd year) and senior (4th year) nursing students experience a critical transitional period in which education shifts from classroom-centered theoretical learning to clinical practice-focused training [4]. During this phase, students are particularly vulnerable to sleep deprivation and emotional burnout, exacerbated by the pressure of the national licensing examination and uncertainty regarding future career paths [2]. These cumulative academic and clinical demands intensify over time, leading to a progressive deterioration in QoL among nursing students balancing clinical rotations and academic responsibilities [2,5].

Furthermore, distinct biological and behavioral vulnerabilities exacerbate these challenges. As the majority of nursing students in Korea are female, they are exposed to cyclic physiological stressors such as premenstrual syndrome (PMS), which can compound daily stress [2,6].

Health-promoting lifestyle (HPL) factors, including physical activity and stress management, constitute essential components of nursing students’ well-being but are frequently insufficient in this population [7]. These findings suggest that modifiable health behaviors play a particularly important role in nursing students’ QoL.

Self-determination (SDT), defined as the autonomous regulation of behavior through the satisfaction of autonomy, competence, and relatedness [8], is a critical psychological factor influencing QoL [9,10]. Meta-analytic evidence supports this, showing that the satisfaction of these basic psychological needs is positively associated with life satisfaction and health behaviors, while negatively correlated with depression [9,10]. Within the nursing domain, SDT frameworks have proven valuable not only for understanding patient support [11] but also for enhancing student development. SDT is a significant predictor of college adjustment among university students [12] and is linked to higher self-esteem, professional identity, and QoL in nursing stu-dents [13]. Furthermore, it positively influences academic achievement and stress management [14]. Notably, a study of Korean nursing students identified competence and relatedness as key predictors of life satisfaction [14]. Given the high academic and clinical demands placed on Korean nursing students [13], this study examines SDT-based SDT as a primary determinant of their QoL [9–13].

PMS is a cyclic condition characterized by physical and psychological symptoms that impair daily functioning and QoL and may also adversely affect academic productivity and clinical practice [15,16]. Previous studies suggest that PMS is a key factor associated with QoL among nursing students. For example, PMS was identified as the most influential factor associated with QoL among Korean nursing students [15], and a study of Brazilian nursing students reported substantial interference of PMS symptoms across major life domains, including family relationships, social activities, and academic efficiency [17]. Consistently, a significant negative correlation between PMS severity and health-related QoL has been documented in female nursing students [15]. Such associations extend to the clinical setting, where PMS is linked to poorer work-related QoL among professional nurses [18].

HPL, encompassing physical activity, nutrition, stress management, health responsibility, interpersonal relationships, and spiritual growth, is a fundamental determinant of QoL for nursing students [19]. Previous research has consistently shown that greater engagement in health-promoting behaviors is associated with better QoL in this population [20,21]. Despite its significance, nursing students frequently exhibit insufficient engagement in health behaviors, particularly reporting the lowest levels in physical activity due to academic burdens [7,21]. Therefore, assessing HPL is essential to identify actionable factors for enhancing the QoL of these future health professionals.

Therefore, this study aimed to identify factors associated with the QoL of Korean female nursing students, focusing on SDT, PMS, and HPL. The study assessed the levels of SDT, PMS, HPL, and QoL, examined correlations among these variables, and identified factors associated with QoL among Korean female nursing students. The findings may provide empirical evidence to inform the development of educational and supportive strategies to enhance students’ QoL and well-being during nursing education.

### 2. Study aim

This study aimed to identify the factors associated with QoL among female nursing students by examining key psychological (e.g., SDT, including autonomy, competence, and relatedness) [8], physiological (e.g., PMS and menstrual characteristics) [2,15], HPL (e.g., health-promoting behaviors such as physical activity and stress management) [19], and academic-related characteristics. Specifically, the study sought to (1) assess levels of SDT, PMS, HPL, and QoL, (2) examine relationships among these variables, and (3) identify factors significantly associated with QoL. The findings are expected to inform the development of educational and support strategies to enhance the well-being and academic adaptation of nursing students.

## METHODS

### 1. Study design

This study employed a cross-sectional descriptive correlational design to investigate factors influencing QoL among female nursing students. The study was reported in accordance with the Strengthening the Reporting of Observational studies in Epidemiology guidelines.

### 2. Participants

The participants were female nursing students in their junior (3rd year) or senior (4th year) year who were undertaking clinical practice at a Presbyterian Medical Center (PMC) in Jeollabuk-do, Korea. They were recruited from nine nursing colleges that operated clinical practicum courses at this hospital. The inclusion criteria were as follows: female nursing students in the 3rd or 4th year of study, aged < 40 years; currently undertaking clinical practice; able to read and understand the study information and questionnaire; and voluntarily agreeing to participate by providing written informed consent. The exclusion criteria were nursing students who declined to participate and those who were receiving psychiatric counseling or pharmacological treatment for mental health problems or mood disorders.

The sample size was calculated using the G*Power 3.1.9.7 program (Heinrich Heine University, Düsseldorf, Germany) for multiple regression analysis with a significance level (α) of .050, a statistical power (1−β) of .95, a medium effect size (f² = .15) [15], and 12 predictor variables (age, grade, body mass index [BMI], menstrual quantity, menstrual cycle, use of analgesics, satisfaction with nursing major, satisfaction with school life, satisfaction with economic status, SDT, PMS, and HPL). The minimum required sample size was 210 participants. Considering potential non-response or incomplete questionnaires, 210 students were recruited. All 210 ques-tionnaires were returned; however, four were excluded due to patterned or inconsistent responses, and data from 206 participants were included in the final analysis.

### 3. Instruments

Based on previous studies [7,10,15], factors expected to influence QoL among female nursing students included SDT, PMS, HPL, and QoL itself. All instruments were used with permission from the original developers.

#### 1) SDT

SDT was measured using the Basic Psychological Needs Scale (BPNS), originally developed by Ryan and Deci [22] and translated and validated into Korean by Lee and Kim [23]. The BPNS assesses the three basic psychological needs proposed in SDT theory: autonomy, competence, and relatedness. The instrument comprises 18 items (6 items per subdomain), each rated on a 5-point Likert scale ranging from 1 (not at all) to 5 (very much). Total scores range from 18 to 90 (possible range: 18–90), with higher scores indicating a greater level of SDT. Subscale scores range from 6 to 30 for autonomy, competence, and relatedness, respectively (possible range: 6–30 for each subscale). At the time of development, Cronbach’s α values for the Korean version were .84 for autonomy, .88 for competence, and .86 for relatedness [23]. In this study, Cronbach’s α for the total scale was .87.

#### 2) PMS

PMS was assessed using the Menstrual Distress Questionnaire (MDQ), originally developed by Moos [24]. The Korean language version provided by the copyright holder, Mind Garden (https://www.mindgarden.com/), was used after purchasing the license. The MDQ consists of 47 items rated on a 5-point Likert scale from 1 (not at all) to 5 (very much). Total scores range from 47 to 235 (possible range: 47–235), with higher scores indicating greater severity of pre-menstrual symptoms. Cronbach’s α for the original instrument was .95 [24], and Cronbach’s α in this study was also .95.

#### 3) HPL

HPL was measured using the Korean version [25] of the Health Promoting Lifestyle Profile II (HPLP-II), originally developed by Walker, Sechrist, and Pender [26]. Permission to use the instrument was obtained from the original authors via e-mail. The HPLP-II comprises 52 items rated on a 4-point Likert scale from 1 (not at all) to 4 (regularly). Total scores range from 52 to 208 (possible range: 52~208), with higher scores indicating a higher level of health-promoting behaviors. The instrument includes six subdomains with the following item numbers and possible score ranges: spiritual growth (9 items, possible range: 9~36), physical activity (8 items, possible range: 8~32), health responsibility (9 items, possible range: 9~36), interpersonal relations (9 items, possible range: 9~36), nutrition (9 items, possible range: 9~36), and stress management (8 items, possible range: 8~32). Cronbach’s α was .92 in the original scale [26], .93 for the Korean version [25], and .95 in the present study.

#### 4) QoL

QoL was assessed using the Korean version of the World Health Organization QoL assessment instrument, abbreviated version (WHOQOL-BREF), derived from the WHOQOL-100 [27] and standardized in Korean by Min et al. [28]. Permission to use the WHOQOL-BREF was obtained from the original developers via e-mail. The WHOQOL-BREF consists of 26 items, including two items assessing overall QoL and general health and 24 items covering four domains: physical health (7 items, possible range: 7~35), psychological health (6 items, possible range: 6~30), social relationships (3 items, possible range: 3~15), and environment (8 items, possible range: 8~40). Each item is rated on a 5-point Likert scale from 1 (not at all) to 5 (very much). Three negatively worded items (items 3, 4, and 26) were reverse scored. Total scores range from 26 to 130 (possible range: 26~130), with higher scores indicating better QoL. Cronbach’s α was .96 at the time of the original instrument’s development [27], .89 for the Korean version [28], and .93 in this study.

### 4. Data collection

Data was collected from July 1 to August 19, 2024. The target population consisted of female nursing students under 40 years of age who were enrolled as junior (3rd year) or senior (4th year) students at nine nursing colleges and were undertaking clinical practice at a PMC in Jeollabuk-do, Korea. Prior to data collection, the researcher contacted the nursing departments of each college, explained the purpose and procedures of the study, and obtained permission and cooperation.

After institutional permission was obtained, recruitment notices containing detailed information about the study were posted in nursing students’ locker rooms and lounges at the hospital. The notices included a QR code that directed potential participants to the online survey platform, Google Forms. When accessing the survey, participants first viewed an electronic information sheet describing the study’s purpose, procedures, voluntary nature of participation, assurance of anonymity and confidentiality, the right to withdraw at any time without penalty, and the use of collected data solely for research purposes. Participants who agreed to these conditions provided online informed consent before proceeding with the questionnaire.

The self-administered online questionnaire took approximately 20 minutes to complete. To acknowledge their time and participation, each student who completed the survey received a small incentive in the form of a mobile gift certificate valued at approximately 5,000 KRW (3.41 USD).

### 5. Data analysis

Data was analyzed using SPSS/WIN version 26.0 (IBM Corp., Armonk, NY, USA). Descriptive statistics (frequencies, percentages, means, and standard deviations) were used to summarize participants’ general characteristics and the main study variables (SDT, PMS, HPL, and QoL). Differences in QoL according to general characteristics were examined using independent-samples t-tests and one-way analysis of variance (ANOVA), and Scheffé post hoc tests were performed for variables showing significant group differences in ANOVA. Pearson correlation coefficients were calculated to examine relationships among SDT, PMS, HPL, and QoL. Skewness and kurtosis values were inspected to confirm approximate normality, and the internal consistency of each instrument was evaluated using Cronbach’s α.

To identify factors associated with QoL, multiple linear regression analysis was performed using the enter method, in which all selected predictors were entered simultaneously in a single block. Ten variables that showed significant associations with QoL in univariate analyses were entered simultaneously into the regression model: three main predictors (SDT, PMS, and HPL) and seven general characteristics (age, grade, menstrual quantity, menstrual cycle regularity, satisfaction with nursing major, satisfaction with school life, and satisfaction with economic status). Categorical general characteristics were dummy coded. Prior to the regression analysis, the Durbin–Watson statistic (2.06) was used to assess autocorrelation of residuals and indicated no serious autocorrelation. Multicollinearity among independent variables was evaluated using tolerance and variance inflation factor (VIF) values; tolerances ranged from .37 to .90 and VIF values ranged from 1.12 to 2.74, indicating no concerns regarding multicollinearity. A two-sided *p*-value of < .050 was considered statistically significant.

### 6. Ethical considerations

To ensure the ethical protection of the participants, this study was conducted in accordance with the principles of the Declaration of Helsinki and was approved by the Institutional Review Board of PMC (No. PMS05-017). Eligible nursing students accessed the study via a QR code, reviewed an online information sheet that described the study purpose, procedures, minimal risk, voluntary nature of participation, and the right to withdraw at any time without any disadvantage, and then provided electronic informed consent before completing the questionnaire. No personally identifiable information (e.g., name, student ID number, or contact information) was collected, and all responses were anonymized and managed using study-specific identification codes only. The data will be stored securely in password-protected files for 3 years after completion of the study and will then be permanently destroyed.

## RESULTS

### 1. General characteristics of participants

Most participants were aged 20~24 years (76.2%) and were senior students (62.6%). Regarding physical and menstrual characteristics, 59.7% of participants were in the normal weight category (BMI 18.5~24.9 kg/m²) [29], 64.6% reported an average menstrual quantity, and 68.9% had regular menstrual cycles. In terms of satisfaction, the highest rates were observed for economic status (85.0%) and nursing major (81.6%). Table 1 presents the differences in QoL according to these general characteristics. QoL scores were significantly higher among participants aged 20~24 years (F = 3.16, *p* = .045), senior students (t = −2.86, *p* = .005). With respect to menstrual health, significant differences were found in QoL based on menstrual quantity (F = 3.06, *p* = .049) and cycle regularity (t = −2.19, *p* = .031), with those reporting regular cycles demonstrating higher QoL. Furthermore, satisfaction with nursing major (t = 5.24, *p* < .001), school life (t = 7.28, *p* < .001), and economic status (t = 5.79, *p* < .001) were significantly associated with QoL. No significant differences were found regarding BMI or analgesic use.

### 2. Levels of SDT, PMS, HPL, and QoL

Table 2 presents the descriptive statistics for the major study variables. The mean QoL score was 92.84 ± 14.81 (possible range: 26~130). Regarding the predictor variables, the mean score for SDT was 70.22 ± 7.20. The mean scores for PMS and HPL were 132.05 ± 31.44 and 130.56 ± 24.18, respectively.

### 3. Correlations among SDT, PMS, HPL, and QoL

Table 3 presents the correlations among the study variables. QoL exhibited significant positive correlations with HPL (r = .63, *p* < .001) and SDT (r = .55, *p* < .001), while demonstrating a significant negative correlation with PMS (r = −.42, *p* < .001). Regarding the relationships among predictor variables, SDT was positively associated with HPL (r = .52, *p* < .001) and negatively associated with PMS (r = −.20, *p* < .001). Additionally, a weak negative correlation was observed between HPL and PMS (r = −.15, *p* = .032).

### 4. Factors influencing QoL

Multiple linear regression was conducted to identify the factors influencing QoL (Table 4). The model was statistically significant (F = 27.56, p < .001) and explained 61.0% of the variance in QoL (adjusted R² = .61). To assess the assumptions of the regression model, autocorrelation of residuals was examined using the Durbin–Watson statistic (2.06), which indicating no serious autocorrelation. Multicollinearity was evaluated using variance inflation factors (VIFs) and condition indices; VIF values ranged from 1.12 to 2.74 and all condition indices were below 30, indicating no concerns regarding multicollinearity. HPL showed the strongest positive association with QoL (β = .37, *p* < .001), followed by SDT (β = .24, *p* < .001). In contrast, PMS was negatively associated with QoL (β = −.21, *p* < .001). Among covariates, satisfaction with economic status (satisfied vs. dissatisfied) was positively associated with QoL (β = .16, *p* = .001), and average menstrual quantity (average vs. large) was associated with higher QoL (β = .13, *p* = .028). Satisfaction with the nursing major (satisfied vs. dissatisfied) was negatively associated with QoL (β = −.11, *p* = .035). Other demographic and menstrual characteristics were not significantly associated with QoL in the adjusted model (*p* > .050).

## DISCUSSION

This study examined factors associated with QoL among nursing students using multiple linear regression analysis. The final model accounted for 61% of the variance in QoL (adjusted R² = .61). In the multivariable model, HPL showed the largest positive association with QoL, followed by SDT, satisfaction with economic status, and menstrual quantity (average vs. large), whereas PMS and dissatisfaction with the nursing major were negatively associated with QoL. Taken together, these findings indicate that nursing students’ QoL is related to multiple domains, including behavioral habits (HPL), psychological resources (SDT), and reproductive health-related factors (PMS and menstrual quantity). Given the cross-sectional design, these findings should be interpreted as associations.

HPL was the strongest positive predictor of QoL in our model. This finding indicates that modifiable health behaviors are a key factor associated with QoL among female nursing students, potentially contributing more to QoL variation than other individual and contextual variables included in the analysis. However, previous research indicates that despite possessing professional health knowledge, nursing students often exhibit poor lifestyle habits such as low levels of physical activity and irregular dietary patterns due to heavy academic burdens and the constraints of clinical practicums [7,20]. Given that HPL is effective in mitigating negative physical and psychological states, such as fatigue and depression [21], active engagement in health behaviors is essential for maintaining QoL. Therefore, the strong association between HPL and QoL observed in this study underscores the need for nursing education to extend beyond theoretical instruction. Both curricular and extracurricular programs should be designed to include integrated, actionable strategies—such as stress management workshops and accessible physical activity routines—that facilitate the adoption and sustainability of health-promoting behaviors within the demanding academic environment [20,30].

SDT was identified as the second strongest positive predictor of QoL. Within the WHOQOL framework, QoL is defined as a broad, multidimensional concept reflecting an individual’s perception of their position in life [3]. This finding corroborates recent research involving nursing students, which identified SDT as a primary determinant of life satisfaction [14]. Theoretically, SDT posits that the satisfaction of three basic psychological needs (autonomy, competence, and relatedness) fosters intrinsic motivation, thereby enhancing subjective well-being [8,22].

In the context of nursing education, characterized by high-pressure clinical rotations and rigorous academic demands, higher levels of SDT likely function as a psychological buffer. These internal resources enable students to perceive academic challenges not merely as burdens but as opportunities for professional development. Consistent with meta-analytic evidence demonstrating that SDT-informed interventions effectively improve both health behaviors and psychological outcomes [10], these findings suggest that nursing curricula should incorporate strategies to foster students’ autonomy and competence (e.g., providing rationale for tasks and acknowledging students' perspectives) alongside clinical training. However, given the cross-sectional design of this study, these interpretations should be understood as discussing associations rather than confirming causal relationships.

From a basic nursing science perspective, the results indicate that reproductive health variables are significant correlates of QoL. PMS was identified as a significant negative predictor of QoL. This finding corroborates previous research reporting that among menstrual attitudes, stress responses, and PMS, PMS was the most influential negative factor on QoL [15]. Similarly, this aligns with studies demonstrating that high levels of stress and PMS significantly compromise QoL [2].

Regarding menstrual characteristics, participants reporting an ‘average’ menstrual quantity exhibited significantly higher QoL compared to those reporting a ‘large’ quantity. Previous re-search indicates that characteristics such as heavy menstruation and irregular cycles exacerbate PMS severity [31]. Notably, the mean PMS score in this study was 132.05, which is relatively high. This elevation may be related to the high levels of daily life and clinical practicum stress commonly reported among nursing students, as these stressors have been shown to significantly increase PMS severity [32].

Consequently, these findings support the necessity of integrated reproductive health interventions. Practical strategies should encompass education on menstrual cycle regulation and pain management, as well as the establishment of medical referral systems for severe symptoms. Such interventions are crucial not only for current academic performance but may also help pre-vent the deterioration of work-related QoL among future nurses [18,33].

Satisfaction with economic status and the nursing major were also identified as significant predictors of QoL. The positive association between perceived economic stability and QoL is consistent with prior research [3,6], suggesting that financial burdens related to tuition and living expenses may act as significant stressors, thereby diminishing students' well-being. Furthermore, the relationship between satisfaction with one's major and higher QoL supports the finding that academic satisfaction is a critical determinant of happiness and well-being among nursing students [34].

This study has several limitations. First, the cross-sectional design precludes causal inference, and the recruitment of participants from a single region limits the generalizability of the findings. Second, as data collection occurred during the vacation period, the reduced academic burden compared to the regular semester may have contributed to relatively higher reported QoL scores. Third, the reliance on self-report instruments introduces the potential for response bias. Specifically, social desirability bias, whereby participants may overreport positive health behaviors, and subjective interpretations cannot be excluded. Although anonymity was assured to encourage honest reporting, future studies should incorporate objective indicators (e.g., physiological measures) to complement self-reported assessments. Additionally, future research should employ longitudinal designs to track QoL fluctuations across the academic semester and recruit geo-graphically diverse samples.

Despite these limitations, this study is meaningful in demonstrating that nursing students’ QoL is associated with a multi-domain set of factors, including modifiable health behaviors (HPL), psychological resources (SDT), and reproductive health–related symptoms (PMS and menstrual characteristics). These factors reflect core aspects of routine health assessment and health management commonly addressed in nursing education and practice. The strong role of HPL identified in this study is consistent with prior evidence highlighting the contribution of health-promoting behaviors to QoL among nursing students [7] and their associations with fatigue and depression-related outcomes [21]. In addition, the observed association between SDT and QoL aligns with nursing student research linking SDT to life satisfaction [14] and with meta-analytic evidence indicating that SDT-informed interventions can improve health behaviors and psycho-logical outcomes [10]. Finally, the negative association between PMS and QoL is supported by evidence among Korean nursing students identifying PMS as a prominent correlate of QoL [15]. Collectively, these findings support the integration of systematic health assessment, health-promotion education, and self-management support into nursing curricula, providing a foundation for student well-being and future patient care. Furthermore, they provide empirical support for developing integrated student support strategies that strengthen feasible HPL practices within training constraints, incorporate autonomy and competence supportive educational approaches, and implement systematic screening and supportive management for PMS-related symptom burden.

## CONCLUSION

Understanding factors associated with QoL is essential for identifying nursing students at risk for reduced well-being and for developing tailored support within nursing education. This study found that higher HPL and SDT, greater satisfaction with economic status, and average (vs. large) menstrual quantity were associated with higher QoL, whereas greater PMS and dissatisfaction with the nursing major were associated with lower QoL. From a biological nursing perspective, this study underscores that physiological factors such as PMS and menstrual characteristics, together with SDT and HPL, play a critical role in shaping the QoL of nursing students. These findings support integrated student-support strategies that strengthen HPL and SDT within the constraints of academic coursework and clinical practicums, while providing systematic assessment and supportive management for PMS-related symptom burden and linking students to resources addressing economic strain and major related concerns. Given the cross-sectional design, the findings should be interpreted as associations, and future studies using longitudinal designs and objective indicators are warranted to clarify temporal relationships and evaluate targeted interventions.

## Figures and Tables

**Table 1.** Differences in Quality of Life According to Participants’ General Characteristics (N = 206)

Variables	Categories	n (%)	M ± SD	t or F (*p*) / Scheffé
Age (years)	20–24^a^	157 (76.2)	93.90 ± 13.79	3.16 (.045)
	25–29^b^	32 (15.5)	92.00 ± 18.34	c < a
	≥ 30^c^	17 (8.3)	84.59 ± 14.80	
Year in academic program	Junior	77 (37.4)	89.09 ± 14.96	–2.86 (.005)
	Senior	129 (62.6)	95.08 ± 14.32	
BMI (kg/m²)	< 18.5	36 (17.5)	93.33 ± 14.95	1.06 (.368)
	18.5–24.9	123 (59.7)	93.74 ± 14.65	
	25.0–29.9	43 (20.9)	90.79 ± 15.60	
	≥ 30.0	4 (1.9)	82.75 ± 4.79	
Menstrual quantity	Small^a^	35 (17.0)	92.94 ± 13.73	3.06 (.049)
	Average^b^	133 (64.6)	94.30 ± 14.66	
	Large^c^	38 (18.4)	87.63 ± 15.52	
Menstrual cycle	Irregular	64 (31.1)	89.27 ± 16.58	–2.19 (.031)
	Regular	142 (68.9)	94.45 ± 13.70	
Analgesic use	Yes	83 (40.3)	94.04 ± 14.70	–0.95 (.342)
	No	123 (59.7)	92.03 ± 14.89	
Satisfaction with nursing major	Satisfied	168 (81.6)	95.26 ± 13.99	5.24 (< .001)
	Dissatisfied	38 (18.4)	82.13 ± 13.69	
Satisfaction with school life	Satisfied	110 (53.4)	99.11 ± 12.56	7.28 (< .001)
	Dissatisfied	96 (46.6)	85.66 ± 13.96	
Satisfaction with economic status	Satisfied	175 (85.0)	95.18 ± 13.47	5.79 (< .001)
	Dissatisfied	31 (15.0)	79.65 ± 15.34	

BMI categories were defined according to the WHO “Malnutrition in Women” classification: moderate and severe thinness, underweight (BMI < 18.5 kg/m^2^), normal weight (BMI 18.5–24.9 kg/m^2^), overweight (BMI ≥ 25.0 kg/m^2^), and obesity (BMI ≥ 30.0 kg/m^2^). Superscript letters (^a^, ^b^, ^c^) indicate groups compared using the Scheffé post hoc test. No significant pairwise differences were identified for menstrual quantity. M = Mean; SD = Standard deviation; BMI = Body mass index.

**Table 2.** Levels of Self-determination, Premenstrual Syndrome, Health-promoting Lifestyle, and Quality of Life (N = 206)

Variables	M ± SD	Min	Max	Possible range
QoL	92.84 ± 14.81	49	129	26–130
SDT	70.22 ± 7.20	54	88	18–90
PMS	132.05 ± 31.44	57	217	47–235
HPL	130.56 ± 24.18	69	200	52–208

M = Mean; SD = Standard deviation; Min = Minimum; Max = Maximum; QoL = Quality of life; SDT = Self-determination; PMS = Premenstrual syn-drome; HPL = Health-promoting lifestyle.

**Table 3.** Correlations among Self-determination, Premenstrual Syndrome, Health-promoting Lifestyle, and Quality of Life (N = 206)

Variables	QoL	SDT	PMS
	r (*p*)		
SDT	.55 (< .001)	1	-
PMS	−.42 (< .001)	−.20 (< .001)	1
HPL	.63 (< .001)	.52 (< .001)	−.15 (.032)

Values are Pearson correlation coefficients (r) with p values in parentheses. QoL = Quality of life; SDT = Self-determination; PMS = Premenstrual syndrome; HPL = Health-promoting lifestyle.

**Table 4.** Factors Influencing Quality of Life among Nursing Students (N = 206)

Variables	B	SE	β	t	*p*	95% CI		VIF
						Lower	Upper	
(Constant)	31.36	9.00		3.49	.001	13.61	49.11	
Age 1 (20–24 years) (ref. ≥ 30 years)	2.03	2.51	.06	0.81	.419	−2.92	6.98	2.74
Age 2 (25-29 years) (ref. ≥ 30 years)	-2.08	2.93	−.05	−0.71	.479	−7.85	3.69	2.70
Year in academic program (senior) (ref. junior)	2.73	1.41	.09	1.93	.055	−0.06	5.52	1.12
Menstrual quantity 1 (small) (ref. large)	3.05	2.29	.08	1.34	.184	−1.46	7.60	1.77
Menstrual quantity 2 (average) (ref. large)	3.94	1.78	.13	2.21	.028	0.43	7.45	1.74
Menstrual cycle (regular) (ref. irregular)	0.15	1.48	.01	0.10	.917	−2.76	3.07	1.12
Satisfaction with major (satisfied) (ref. dissatisfaction)	− 4.00	1.88	−.11	−2.13	.035	−7.71	−0.29	1.28
Satisfaction with school life (satisfied) (ref. dissatisfaction)	−2.35	1.55	−.08	−1.51	.132	−5.42	0.71	1.44
Satisfaction with economic status (satisfied) (ref. dissatisfaction)	6.66	1.96	.16	3.40	.001	2.80	10.52	1.18
Self-determination	0.50	0.11	.24	4.50	< .001	0.28	0.72	1.54
Premenstrual syndrome	−0.10	0.02	−.21	−4.42	< .001	−0.15	−0.06	1.22
Health-promoting lifestyle	0.22	0.03	.37	6.75	< .001	0.16	0.29	1.58
Adjusted R^2 ^(*p*)	.61 (< .001)							
F (*p*)	27.56 (< .001)							

SE = Standard error; CI = Confidence interval; VIF = Variance inflation factor.

## Data Availability

Please contact the corresponding author for data availability.
